# Mental health problems among conflict-affected adults in Grozny, Chechnya: a qualitative study

**DOI:** 10.1186/s13031-016-0083-8

**Published:** 2016-08-03

**Authors:** Amanda J. Nguyen, Concetta Feo, Kyuri Idrisov, Giovanni Pintaldi, Annick Lenglet, Zalina Tsatsaeva, Paul Bolton, Judith Bass

**Affiliations:** 1 Department of Mental Health, Johns Hopkins School of Public Health, 624 N. Broadway, Baltimore, MD 21205 USA; 2 Médecins Sans Frontières, Moscow, Russia; 3 Department of Psychiatry, Chechen State University, Grozny, Chechen Republic Russian Federation; 4 Public Health Department, Médecins Sans Frontières, Amsterdam, The Netherlands; 5 Center for Refugee and Disaster Response, Department of International Health, Johns Hopkins School of Public Health, Baltimore, USA

**Keywords:** Chechnya, Mental health, Psychosocial, Aggression, Depression

## Abstract

**Background:**

A decade of conflict in Chechnya destroyed infrastructure and resulted in widespread exposure to violence. Amidst substantial reconstruction, periodic violence has contributed to an ongoing atmosphere of insecurity. We conducted a qualitative study to understand the mental health and psychosocial problems affecting adult Chechens in this context to inform development of assessment tools for an evaluation study related to individual counseling.

**Methods:**

Data were collected in July 2014. A convenience sample of 59 Chechen adults was asked to Free List all problems affecting people in the area. Four problems were explored further in 19 Key Interviewee (KI) interviews, with respondents identified using snowball sampling. Data analysis was conducted in Russian by the Chechen interviewers.

**Results:**

Multiple mental health and psychosocial problems emerged, including ‘bad psychological health’, ‘depression’, ‘stress and nervous people’, and ‘problems in the family’. Aggression, ‘emotional blowing’, and ‘not adequate’ behavior were frequently reported indicators of these problems, with negative effects on the whole family. Chechens reported seeking help through informal social networks, psychiatric and psychological services, and Islamic Centers.

**Conclusion:**

Chechens reported mental health and psychosocial problems similar to those experienced in other post-conflict settings. The description of ‘emotional blowing’ mirrored prior findings in Chechen asylum seekers and fits within a cluster of cultural concepts of distress featuring anger that has been identified in other conflict-affected populations. Further exploration of the nature and prevalence of this construct, as well as evaluations of interventions aimed at reducing these symptoms, is warranted.

## Background

The mental health impacts of conflict have been well-documented, with reviews consistently citing elevated rates of mental disorders in populations exposed to conflict and other mass violence [1, 2]. Risk of disorder in a given context is influenced by both individual and situational factors, such as level of exposure to potentially traumatic events, time since conflict, and level of displacement [3]. Beyond the direct effects of mass violence, mental health is also affected through prolonged negative impacts of violence on economic development, social capital, human rights, and the availability of supportive resources [4, 5]. Additionally, cultural factors influence mental health by impacting what is interpreted as a traumatic event, how community norms and practices elevate or protect against risks to mental health, and how mental distress itself is expressed [6, 7]. Much of the research on mental health in conflict has focused predominantly on PTSD, thus we know less about other mental health problems that may be prominent in communities affected by long-term repercussions of conflict [8].

In the Republic of Chechnya in the North Caucasus, the mental health needs of the local population are not well understood. Chechnya is an autonomous region in Southern Russia with a population of approximately 1.2 million people, bordered by Russia and Ingushetia to the North and West, and Georgia and Dagestan to the South and East [9]. Ethnic Chechens comprise the majority of the population and are predominantly Sunni Muslims whose religious practices incorporate Sufism and pre- Islamic beliefs [10]. Society is patriarchal and structured around a clan system with a clear hierarchy within the family [10]. Chechens have a strong ethnic identity [11]; customs and traditions follow a social code, *Nokhchallah*, which is described as “Chechenness—the quality of being a Chechen” [10, p. 129]. The social code places high value on individual duty, family honor, respect for women, hospitality, friendship and brotherhood, and mutual help and charity [10, 12].

Following its declaration of independence from the Soviet Union in 1991, Chechnya has experienced continued instability. From 1994–1996, and again in 1999, the population experienced repeated bombing, heavy fighting with widespread exposure to violence, displacement, and destruction of services and infrastructure [13]. The death toll from the two conflicts has been estimated at 160,000 people [14], and the humanitarian impact of these events have been well-documented [15, 16]. While the region has undergone massive reconstruction and security has improved in recent years, periodic violence has sustained a general atmosphere of insecurity [9]. Health and social service systems remain overburdened and underequipped to meet the needs of the community [17]. The conflict disrupted education systems and resulted in massive brain-drain as many of the most educated left the republic [18]. The economy, which prior to the conflicts had been based on oil production and agriculture, has been bolstered by aid but has struggled to recover, resulting in an unemployment rate double that of the surrounding areas [18].

Most of what is known about the mental health and psychosocial needs of the Chechen people is based on a small number of studies conducted with refugees and internally displaced people at the height of the conflict. A 2004 study of 539 displaced Chechens showed that nearly all had been confronted with violence; a third had directly experienced war-related violence, and nearly a quarter had witnessed the killing of a person. Using a General Health Questionnaire cut-off score of 11, approximately 80 % of those interviewed were suffering from significant health complaints, including somatic complaints, symptoms of anxiety or depression, and social dysfunction; however, the GHQ cutoff was not validated in this context, so these findings must interpreted with caution [19]. In an evaluation of 50 Chechen asylum seekers located via convenience sampling in Austria, 62 % showed clinically significant trauma symptoms as assessed by clinicians [20]. A study of Chechen youth in temporary camps also reported higher internalizing problems when compared with non-conflict- affected Russian youth [21]. The same study of Chechen youth also reported a variety of physical and emotional stressors related to displacement, including worry about the future and feelings of humiliation [22]. Yet given the changing social context in Chechnya over the past decade and the potential differences between asylum seekers and those who stay, the above studies cannot be assumed to represent the current mental health needs of people in the Republic.

The international medical organization *Médecins Sans Frontières* (Doctors without Borders; MSF) has been providing individual mental health counseling in Chechnya since 2003. In preparation of an outcome evaluation of the MSF mental health program’s counseling approach, we undertook a brief qualitative study to improve our understanding of current mental health problems experienced by Chechen adults presently living in Chechnya, including the nature of the problems and locally relevant coping and help-seeking behaviors. The primary purpose of this study was to provide data to develop and adapt culturally valid assessment instruments for use in the outcome evaluation. These findings also have broader implications for improving our understanding of mental health needs in contexts of prolonged insecurity after the resolution of the acute phase of conflict.

## Methods

### Study site

Data were collected in the Chechen capital city of Grozny, Chechnya’s largest city and administrative center, over a three-week period in July 2014. The city was a site of heavy fighting and military bombardment during both Chechen conflicts, resulting in almost total destruction [23]. Grozny has also been a major focus of reconstruction efforts, including new roads, hospital and university buildings, a regional airport, and a new mosque. The study was undertaken by MSF in collaboration with Chechen State University.

### Participants

Study participants included 78 Chechen adults from multiple sites in downtown Grozny, including hospital and community locations. Free List (FL) interviews (24, p. 32) were conducted with a convenience sample of three types of respondents: 1) MSF counseling clients (*n* = 12); 2) hospital patients receiving treatment (on either an in-patient or out-patient basis) for issues other than mental health problems (*n* = 14); and 3) general community members (*n* = 33). We purposively sampled to have gender balance for each respondent type and to capture a range of ages (18–67 years, *mean* = 40 years). The rationale for including a small number of counseling and non-mental health hospital patients along with general community members was to ensure that perspectives of key MSF service groups were included in the study; this was deemed relevant because the key study aims focused on improving assessment of mental health problems patients were likely to seek treatment for at the MSF counseling program. No compensation was provided to FL respondents.

Following the FL interviews, in-depth interviews (24, p. 42) were conducted with 19 Key Interviewees (KIs) considered by community members to be knowledgeable about mental health problems. KIs were provided 450 rubles, the equivalent of 10 US dollars, as compensation for their time, with the expectation that they be available for two interview sessions lasting one hour each.

### Data collection

Thirteen Chechen interviewers were trained by the study lead (AN) in research ethics, the two qualitative interviewing methods, and data analysis. These interviewers first conducted FL interviews to identify problems affecting people in the area. From the list of problems, potential mental health problems were identified (see analysis below) for further exploration using the KI interviews.

Interviewers worked in teams of two to conduct and record interviews verbatim in Russian, the official language used regularly in the public sphere. Many Chechens speak both Chechen and Russian, often mixed within a single conversation. In cases where a respondent preferred to provide an answer in Chechen the interview team recorded notes in Russian, as this is the written language used by most of the population. When the interview was conducted in Chechen, the Russian translation was confirmed by the interviewee. The study protocol was developed based on the Johns Hopkins University Applied Mental Health Research Group’s Design, Implementation, Monitoring, and Evaluation (DIME) research model, Module 1 [24]. Previous studies in diverse settings such as Haiti [25] and Georgia [26] have demonstrated the utility of this approach for identifying locally relevant mental health problems.

Adult men and women were recruited for FL interviews from MSF counseling services, hospital wards, and in public parks and markets, using convenience sampling. While there were no explicit exclusion criteria for the FL respondents, the nature of the locations required that respondents were reasonably mobile.

Free list interviews (24, p. 32) began with a single open-ended question designed to elicit responses in the form of a list. The purpose of these interviews was to gain a sense of the relative priorities of problems in the population and to identify problems – in the respondents’ own words - to be investigated in the KI interviews. Participants were asked: “What are all the problems affecting people in this area?” The interviewers probed until the respondent could list no more problems. Before ending the interview, the interviewers reviewed the problem list to identify potential mental health problems defined as problems related to *thoughts*, *feelings* or *relationships*. If any such problems were identified, they asked the respondent to suggest people in the community who were knowledgeable about this problem, or whom people would go to for help with this problem. If the respondent suggested someone, the interviewer asked permission to contact this person and recorded his or her name and contact information in a separate notebook. These names formed the initial list of potential Key Interviewees (KIs).

Following the analysis of the FL data (described below), KI interviews were undertaken to gain a deeper understanding of the selected problems (24, p. 42). The first seven KIs were those that were identified during the FL interviews, with subsequent KIs identified through snowball sampling. The initial inclusion criteria were that KIs were Chechens currently living in Chechnya and were identified by the FL respondents as being knowledgeable about the selected mental health problems. Most of the initial KIs were counselors or psychologists. After the first KIs were interviewed, we asked the KIs to refer us to additional KIs who were *not* counselors or psychologists, to ensure that respondents represented a wide set of experiences and backgrounds. KI roles and professions ranged from counseling, teaching, journalism, social services, and service users or family members. KIs were contacted by the study assistant, provided with a brief overview of the study, and invited to participate in an interview at Chechen State University, their home or their office.

For the KI interviews, the interviewer identified the first priority mental health problem from the FL results and asked the KI to describe everything he or she could about that problem. The KI was allowed to speak freely, without interruption, while the interviewers recorded responses verbatim. When the KI stopped, the interviewers asked follow-up questions regarding the nature of the problem, perceptions of causes, the effects of the problem on self and others, and current coping and help-seeking behaviors. After exhausting responses to the first mental health problem, the interviewer proceeded with the next priority problem, until all four selected mental health problems had been fully explored. The interviewers were instructed to end an interview session after one hour to avoid fatigue of the interviewers and KI. After each interview, the interviewers returned to the study office and immediately reviewed the transcript with the study supervisors (AN, CF) using a translator. The supervisors provided coaching and feedback, including specific items to follow-up on and additional questions to ask during a follow-up interview. The interviewers contacted the KIs to set up a second interview the following day. All KI interviews were conducted over a 4-day period and seven KIs (37 %) were available for a follow-up interview during that time.

### Ethics

Respondents were read a standard information sheet about the study and provided either written or verbal consent with a witness (if preferred) prior to being interviewed. A copy of the information sheet in Russian was also made available for respondents to take with them if they chose to do so, with contact information of the study staff. Participant names were not recorded for FL respondents; age, sex, and type of respondent were recorded and used to track the recruitment process. Names and contact information for potential KI respondents were recorded in a separate notebook solely for recruitment and scheduling purposes, and destroyed once interviews were completed. Potential KI respondents were contacted by the study assistant and informed that we had recently interviewed people in the area about problems affecting people here, and that the KI had been recommended as someone who was knowledgeable about a problem we were interested in learning more about. No information was provided about who had referred them.

Interviewers were all current medical students who received training on research ethics and qualitative interviewing methods. Because all questions asked about problems affecting Chechens in general rather than problems respondents had personally experienced, risk of emotional distress due to study participation was expected to be low. Interviewers were trained to redirect any discussion about personal problems or experiences back to problems as experienced by Chechens in general, and to stop an interview and consult their supervisor if a respondent became upset. Supervisors were present on site during FL interviews and available by phone during KI interviews, and met with the interview team following each interview to discuss any problems or concerns that arose. MSF counseling staff were also available to consult by phone if needed. The study protocol was approved by both the MSF and Chechen State University ethical review boards.

### Analysis

All FL and KI transcripts were coded and analyzed in Russian by the Chechen interviewers, with technical assistance from the English-speaking supervisors (AN, CF). FL analysis involved combining all individual FLs into a single master list of problems, ranked by frequency of reporting (Table 1). Decisions as to whether two differently worded items referred to the same problem were made by group consensus. If the interviewers agreed that two items were the same, they selected the better (i.e. more descriptive or representative) of the two wordings to include on the list; if the interviewers could not agree, the items were listed as separate problems. After all lists were combined, the group reviewed the resulting composite list to determine if any items on the list could be combined further, again by consensus. The final master list was reviewed by the study team for potential mental health problems to explore further in the KI interviews. Selection of which problems to select was based on those that were identified by multiple respondents and were also most likely to be addressed by the available MSF counseling program.

**Table 1:** FL problems mentioned by at least three respondents (*n* = 59)

Problem	Frequency	Problem	Frequency
Unemployment	38	Corruption	5
Medical care (quality)	26	The culture has changed	5
No money	25	Divorce	5
Health services (cost)	21	Climate/pollution	5
Problems with education	13	Problems in the family^a^	4
Health problems	13	Drunkenness	4
High prices	13	Depression^a^	4
The ethics of communication	12	Stress/Nervous people^a^	4
Traffic	11	No free speech	4
Low salaries/pensions	10	No social support/assistance	4
Poor behavior of young people	9	Lack of time	3
No roofs over their heads/housing	7	Quality of water	3
Bad psychological health^a^	7	Infertility	3
Drug addiction	7	Religion	3
The young people are not well-bred	6		

^a^Priority problem explored in KI interviews

KI interviews were analyzed similarly to the FL interviews. Interviewer teams reviewed each interview transcript, creating lists of all symptoms, positive and negative effects, and positive and negative coping strategies reported for each of the priority problems. The lists from each KI interview were combined into master lists for each problem.

## Results

### Free lists

A number of mental health related problems emerged from the free list interviews, including: (a) bad psychological health; (b) drug addiction; (c) problems in the family; (d) drunkenness; (e) depression; and (f) stress and nervous people. Commonly reported non-mental health problems represented categories of: (a) service and infrastructure problems such as unemployment and the quality of medical care; and (b) social and relational problems such as changes in culture and communication (see Table 1).

### Key interviewee interviews

Four problems were explored in the KI interviews: 1) *bad psychological health* was selected in order to understand whether this constituted a distinct local syndrome or was a summary term of multiple problems; 2) *depression* and 3) *stress and nervous people* were selected as these are mental health problems the MSF psychosocial counseling program aims to address and intended to evaluate in the subsequent outcome evaluation study; and 4) *problems in the family* was selected to further understand the nature and consequences of these problems. The frequently reported issue of drug and alcohol problems was not selected because substance use was not intended to be assessed as a primary outcome in the subsequent evaluation; however, this problem was often discussed by KIs in the course of their interviews. Below we summarize the features frequently reported for each of the selected problems.

**Bad psychological health** (Table 2) appeared to be a summary term encompassing general distress and an inability to cope with daily stressors: “*For example we have many emotions that we’re not expressing, and one day we can do something bad because of these emotions”.* KIs described this problem as manifesting in verbal and physical aggression (often toward children and family), physical health problems (e.g. *“constant headaches due to nervousness”*), and social withdrawal. A number of KIs talked also about problems with communication: *“people keep everything inside, and this is the tremendous psychological pressure”,* and emphasized that bad psychological health was experienced differently for men and women due to Chechen norms that restrict men from talking about their problems: *“men experience more difficulties than women because they have no place to spill out their emotions…withholding affects mental and physical health, correlate[ing] with poor physical health in Chechen society*” .While some KIs explained that bad psychological health was similar to depression or that depression was a part of bad psychological health, many respondents distinguished this problem from specific disorders. For example, one KI explained, *“a person with bad psychological health can be normal but the emotions will blow up from time to time…if a person just sometimes in the past was inadequate, had emotions blow up, it doesn’t mean he’s sick”.*

**Table 2:** KI descriptions of ”bad psychological health” (n = 19)

Symptom	Frequency
Aggression	7
Physiological diseases	7
Withdrawal and isolation/apathy	5
Tearfulness	4
Problems in communications with others	3
Don’t take care of him/herself	3
Hysterics, screams	2
Loss of appetite	1
Tiredness	1
Distraction	1
Apathy	1
Alcohol	1
Effects	Frequency
Aggressiveness: (nervousness, out of temper, emotional blows, emotional load)	10
Physiological diseases	7
Conflicts within the family (difficulties with relatives and friends)	7
Withdrawal (depressive mood, apathy, indifference, isolation)	5
Alcoholism and drug addiction	4
Problems at work	3
Tearfulness	3
Problems in communications with others	2
Hysterics, crying, screams	2
Don’t take care of him/herself	2
Loss of appetite	1
Tiredness	1
Distraction	1
Apathy	1
Have will to leave and go somewhere	1
Cannot create a family - no money	1
Syndrome of consumers	1
Syndrome of beggar	1
*War (makes person stronger)*	*1*
Positive coping strategies	Frequency
Apply to Psychologists	11
Talk with relatives/friends/close people	7
Rely on Religion	7
Receive treatments (body therapy, meds, rehab centers)	6
To rely on yourself	2
To do some activities (sports, social nets, tv, pc.)	1
Negative coping strategies	Frequency
Refuse help	3
Plunge into work	3
Give up	1

**Depression** (Table 3) was characterized by social withdrawal, functional impairment, and difficulties in communication. One respondent described it as being *“…despondent, when your body refuses to act. Depression is unwillingness of the body to organize something*.” And another: *“It is a hard disease, a heavy disease.”* KIs described people with depression as being indifferent toward themselves and their families, with subsequent effects on personal health and family relationships, saying:

**Table 3:** KI descriptions of “Depression” (*n* = 19)

Symptom	Frequency
Alcohol/drugs	6
Difficulties and stop in communication with people	5
Withdrawal	5
Leads to disability (invalid)	1
Refuse food/drink	1
Effects	Frequency
Suffering within the family (indifference, sense of abandon)	16
Affects the health and physical strength (degradation, indifference)	9
Hopelessness	7
Not adequate behavior	5
Incidents because of lack of concentration (work, daily activities)	5
Egoism	5
Become aggressive	3
Fears	2
*Ameliorations in family*	*2*
*Spirituality and self-esteem*	*1*
Positive coping strategies	Frequency
Apply to the psychologist	13
To talk to your friends, natives, peers	11
To rely on religion (pray, apply Muftiate and Islamic Center, *Belkhi*)	9
Ask for help (doctor, meds, sanatorium, body exercises, rehab)	9
To do activities (sport, diary, tv, music, animals)	6
To rely on yourself	2
To make a career	1
Negative coping strategies	Frequency
Withdrawal	9
Use/Abuse of: energetic drinks/alcohol/drugs/smoke	6
Suicide	3
Antisocial behavior (fighting, join terrorist groups, crimes)	3
To plunge into work	3

*It’s difficult for such people to find mutual language with other people. When he’s at the peak of disease, he’s irritated by his wife, family, children. He’s afraid to go out because he’s afraid he will say something, otherwise he’s afraid of the community. He’s afraid that people will start talking about his disease.*

They described hopelessness and “not adequate” behavior that failed to meet Chechen social norms, including aggression and an inability to look beyond their own problems. One KI illustrated the cognitive and mood components, describing “*Depression is when a person sees in an ugly way everything, in an emotional way.”* Others highlighted the risk of suicide, (e.g.: “*people are doing away with their own lives. Officially it is not recognized, but this is how it is”.*), noting that suicide was viewed as a sin and stigmatized as un-Chechen: *“This is difficult for me to understand – my mentality is Chechen and I still don’t know how that could happen”.*

**Stress and nervous people** (Table 4) featured symptoms of irritability, aggressiveness, and psychological pressure. As one respondent described, *“he is aggressive, irritable, if you tell him something he’s responding in an unethical way. Something has led him to this situation – this is not out of the great life that he became like that”.* KIs also listed a number of associated health problems implicated in prolonged hyper arousal, such as strokes, heart attacks, insomnia, headaches, sweating, and rapid breathing. They repeatedly linked this problem with “not adequate” behavior, “emotional blowing”, and verbal and physical aggression toward themselves and others: *“If a person closes up and doesn’t share, then aggression and depression appear…It’s the form of defense and mentality meaning that people don’t want to be seen as morally weak”.* These concepts were described as being highly associated: *“Particularly in our society, as opposed to the other places, this aggressive behavior is very visible—aggressiveness, tension, nervousness”.*

**Table 4:** KI descriptions of “Stress and nervous people” (*n* = 19)

Symptom	Frequency
Irritability	9
Aggressiveness	7
Apathy and indifference	6
Phobia	4
Tearfulness	3
Alcohol/drug abuse	2
Headache	1
Insomnia	1
Somatic diseases	1
Heart strokes	1
The loss of ability to speak	1
Infarcts	1
Diseases appear	1
Tiredness	1
Confused	1
Cannot concentrate	1
Physiological reactions (sweating, rapid breathing)	1
Effects	Frequency
Psychological symptoms (tearfulness, phobia, hesitancy, no trust, apathy and indifference, withdrawal, confusion)	13
Aggressiveness (physical and verbal: antisocial, yell, anger, rudeness/emotional blows up/not adequate. toward self and others)	11
Psychological pressure (Irritability	9
Conflicts in family and friends (scandals)	6
Physical symptoms (insomnia, tiredness, headache, sweating, rapid breathing, stroke, mutism, infarcts)	5
Negative influence children and youth	3
*Stress resistance*	*3*
Problems at work	2
Migration of community	1
*Progress*	*1*
Positive coping strategies	Frequency
Go to the Psychologist	9
Talk to friends/relatives/advisors	7
Rely on yourself/calm down/sort ideas/patience/rest	5
To do activities (walk, outing, hobbies, gifts)	4
Go to the doc/Neuropathologist/NGO/meds	3
To rely on religion and customs	3
Have children may rescue from depression	2
Negative coping strategies	Frequency
Avoid asking for help	4
Give up (Put hands down)	3
Drugs/alcohol abuse	2
Plunge into work and study	1

**Problems in the family** (Table 5) were described as a cluster of problems including bad family relations, conflicts, jealously, bad communication, divorce, no attention paid toward family, and physical and verbal aggression toward the children and family. Changing gender roles and generational gaps were highlighted, such as: *“a generation grew up abroad, losing tradition and language”.* Problems in the family were also said to impact the community, with increasing criminality and behavior ‘not adequate’ to Chechen norms: *“Problem in the family is bad psychological and physical health of the nation. And the level of criminality and drug addiction increases. And all this comes from the family”.*

**Table 5:** KI descriptions of “Problems in the Family” (*n* = 19)

Symptom	Frequency
Aggressiveness	8
Alcohol/Drugs abuse	4
Effects	Frequency
Bad family relations/conflicts/jealousy/bad communication in family/no attention/divorces)	9
Children development affected (complexes, misbehavior, learning problems, suffering)	8
Aggressiveness (physical, verbal, towards children and family)	7
Psychological pressure (Stress/oppression/weakness/aimless)	5
Drugs/alcohol addiction	4
Bad psycho health (Depression/inadequate behaviors)	3
Negative effects on the community/increasing criminality/Stop being Chechens (the behavior is not adequate to Chechen norms)	3
They go radically into religion	1
Not respect	1
Loss of job	1
Positive coping strategies	Frequency
Go to the Psychologist	10
Talk among natives and relatives/solve problems together	8
Medical center/doctors/NGOs/rehab	7
Rely on yourself/calm down/find interests/rest/outing/compromises	7
Rely on Religion (Mullah/Strength in God) & Traditions (Muftiate/Guardianship)	4
To apply the trainings on sensibilitation	1
Negative coping strategies	Frequency
Postpone problems	6
Plunge on working	2
Take distance from everything	1

#### Causes

In addition to exploring the presentation and description of the selected problems, KIs reported on their perception of the causes of the problems and what people did to help themselves cope or deal with them when they arise. Different problems were not attributed to different causes; rather, multiple causes were discussed as a source of all the explored problems. Among the causes frequently discussed by the KIs were the war and ongoing stressors, changing social norms, and general insecurity. For example: “*people, they are scared of dangerous situations, they always take care of something that they think will happen someday, they explain that they are afraid for their families…they are scared after war that the war will begin again”.* Multiple KIs described an interaction between conflict-related traumatic experiences and proximal stressors that impacted people’s ability to cope: *“Few people receive psychological rehabilitation. A lot of values are lost. And now the reason is social factors, alcoholism and drug addiction.”* As one KI summarized, “*The nature of mankind is so it’s difficult to live under constant tension”,* while others explained: *“Many people suffer. Youth suffer due to lack of self- realization. In the republic, the situation is changing rapidly…So people could not get oriented in their station and build up their life”.*; and:

*Any country who had war, after reconstruction people took out many problems, everything that we cannot explain. So all the things that we can’t explain make us worry about it. We had serious changes after war in religion, in culture…so very sharp changes comparing to the situation before war and after war.*

These changing social structures were also said to impact men and women differently:

*After the war, because of the devastated economy men had no places to work, so women have taken the role of breadwinner. So men being unemployed, losing previous role in the family, falling into depression, start drinking, and then show aggression to the other members of the society.*

And:

*First and foremost, there’s a shift in role between men and women. For instance, the woman is working and she is earning, while the man, for instance, is not working or working with inconsistent income. In such a way there’s a matriarchal society being formed up…in this situation all these things are being changed and there’s a conflict between them, and this is reflected on children.*

In addition to the context of ongoing stress, two KIs talked about the importance of spiritual forces. For example, one KI indicated “*When they have some jinx the symptoms are the same as person that has depression…very weak, no interest to life”.* However, the KIs who spoke about spiritual forces did not appear to view the problems as the same, but rather as separate problems with similar symptom presentation, explaining that there should be a distinction between the two: “*many appeal for help to Islamic centers…but it is not always the case that this is the result of external spirits. These things should be divided, mental health and external forces*”.

#### Negative impacts

A common theme across the described problems – particularly the problems of “bad psychological health” and “stress and nervous people” - was that of aggression, ‘emotional blowing’, and ‘not adequate’ behavior, described as behavior, often aggressive, that deviated from Chechen norms: *“I’m talking about the behavior of people who have stress. It shows up in aggression.” “People become unbalanced and they are spinning off their anger on their children and they beat up their children and shout/yell at them.”* Aggression was also linked to depression, although to a lesser extent:

*“When a person has depression he stops being the person he used to be and spoils relations with friends. And his friends demand from him to be the same as he used to be, but the person is not able to be the same. And here starts aggression. This person needs help but he has only demands from other people. In such case a person turns inside or he starts to be aggressive”.*

KIs repeatedly raised concerns about the effects of this aggression on children, families, and community: “…a*ggression and anxiety. If a woman is beaten, children are afraid, and in the future they take it as a model of behavior and it leads to violence in the family”.* They spoke of aggression displaced onto children: “*a woman who was beating her children, she was very angry at her husband and mother in law but she was beating her children because of that”.* And referred to patterns of behavior affecting their families:

*“The family is about constant relationships. Mentally unhealthy people are breaching this system of the relationships. Mentally unhealthy person is changing his behavior, he becomes passive aggressive, inadequate. He cannot provide any assistance, any help in the solution of the problems of his relatives. If someone is ill in the family, that is being transmitted onto children, directly. As a consequence there are problems with children. There is a family wellbeing that is breached”.*

KIs also identified the impact of this aggression on the community, including the loss of relationships with those manifesting aggression:

“*The aggression of a person will affect everyone who is around him…So we have people who are destroying themselves but it also affects people around because we have really close relationships between relatives. Community lost the part of it faced by this person*”.

#### Coping and help-seeking

Common coping and help-seeking behaviors were similar for all four of the problems explored.

Formal help-seeking behaviors included seeing a psychologist, going to the Islamic center, and seeking medical treatments. Some suggested people suffering from mental health problems would first seek help in primary care: *“Firstly they go to the hospital. If they have a headache, they will go to the doctor and he will think that they have some disease”.* One KI reported women were more likely than men to seek counseling and would present with problems in the family: *“in general, they are women. They go with their home problems, with the problems of gender violence, and with problems of their children”.* Others were said to seek help from religious authorities because *“it’s familiar, that’s why it’s not so scary”.* However, these help-seeking behaviors were not mutually exclusive, and referrals could be made between them. One KI explained, “*When the medicine is helpless people apply to Islamic center. In the Islamic center there are also psychologists.”* KIs reported that the Islamic healers would also refer cases to doctors or psychologists that they were unable to treat using traditional methods, and suggested, “*it is not correct to deny one of these supports, they should work together…Mullah usually gives decisions, but also there should be a way to cope with the problem. And psychologists are giving this opportunity to cope with the problem”.*

Psychologists were described as taking case histories and “*looking for the reasons not applying to the spiritual side”.* Islamic center treatments were described as lectures that support relaxation, treatments using holy water, reading from the Koran, and using oils and herbal treatments. Medical treatments involved body therapy, medication, rehabilitation centers, and respites at sanatoriums abroad: “v*ery frequently people are traveling abroad, because here the treatment is not effective”.* KIs also described that people suffering these problems would seek guidance from the *Muftiat*, or Islamic legal counsel, particularly in the case of family problems. Religion was discussed as a protective factor, including against suicide: *“if not religion, which is the factor that holds people, the quantity of suicides would increase”.*

The most frequently described informal help-seeking strategies involved talking with friends and relatives, or doing self-care activities. One popular activity was going outside to rest; this referred not only to the outdoors but also, in some cases, leaving the Republic either permanently or for holiday: *“going out somewhere for rest is also an important factor.”* Discussing high emigration, some respondents raised concerns about the impact of this on Chechen culture: *“some are trying to go abroad thinking everything is good there, which is not the case. And children growing up there, they cease to be Chechens”.* Negative coping strategies included social withdrawal or refusing help, plunging into work, giving up, antisocial behavior, and substance use. For example, *“Maybe the depression will lead to disability itself. And it leads to drug addiction and to taking psychoactive pills. And the community suffers…and decreases the health of the nation. People are not emotionally resistant”.* The latter not only included mention of alcohol and drugs, but also energy drinks, the use of which is frowned upon in Chechen culture.

## Discussion

In the current study respondents described syndromes similar to depression and a cluster of anxiety/trauma-related symptoms with high levels of aggression and irritability. They also described a more general concept of “bad psychological health,” which appeared to be not only an umbrella term including other symptoms and syndromes but also a psychosocial state that fell somewhere between illness and wellbeing. Although it was not surprising that these syndromes emerged in the FL interviews, the purpose of beginning with free listing was not only to confirm that these were problems of concern but to gain some sense of the relative priorities of problems in the population, and to include the words used by individuals to describe these problems as we explored them further in KI interviews. This approach allowed us to explore syndromes as conceptualized from the local perspective rather than imposing preconceived distinctions between symptoms.

The frequent reports of irritability, aggression, and emotional outbursts associated with mental health problems are similar to findings in a study of asylum seekers from a variety of countries conducted in Austria in the mid-2000s. In that study, conflict-affected Chechens reported higher levels of irritability and aggression compared to West African or Afghan asylum seekers [20, 27]. That these symptoms were also reported by Chechens in the current study suggest that they are not only an immediate response to trauma but perhaps are more typical of the Chechen response regardless of time. Similar accounts of explosive anger and aggression have also been reported in diverse cultures with recent histories of conflict and trauma, such as among East Timorese adults in Southeast Asia, where the symptoms were frequently discussed amidst concerns of violence toward children and families [28]. This suggests that while Chechens may differ from Africans in this response it is not specific to Chechens. Rasmussen and colleagues’ 2014 review of the literature on cultural concepts of distress (CCDs) in trauma-affected populations outside of Europe and North America identified a cluster of concepts distinguished by negative moods and cognitions featuring anger [29]. The prominence of such symptoms in the current study may suggest a relevant cross-cultural (but perhaps not universal) syndrome that fits well within this cluster of CCDs.

The mental health problems identified in our study appeared to be highly interconnected. This is expected in a population that has experienced war with its combination of trauma during hostilities and subsequent deprivations and upheaval, resulting in a wide range of co-occurring mental health problems. Given this, it is not surprising that there was little distinction between problems in terms of the causes that were described. Previous research from multiple trauma-affected populations has suggested that whereas war-related stressors are more closely associated with PTSD-like syndromes, depression and anxiety-like symptoms are often viewed as the result of both past events and current stressors [5, 30] such as those reported in the current study. That we did not identify intrusion and avoidance symptoms characteristic of a PTSD-type syndrome may be because these symptoms are less prominent among Chechens, or not readily reported. Alternatively, it could be that we used a largely community-based sample of respondents, rather than a specifically trauma-affected sample. However, our findings are similar to those of the Rasmussen CCD review [29]. Specifically, they reported that depression-like syndromes emerge much more commonly than PTSD-like syndromes in emic research, that avoidance symptoms are rarely reported, and that across the literature there was no clear distinction between CCDs caused by trauma symptoms rather than chronic stressors [29].

In Chechnya, our findings suggest many of the described proximal causes of current mental health problems are social pressures and chronic stressors rather than explicit trauma exposure. As such, trauma-*informed* treatment programs targeted at symptom reduction, mood regulation, and improved coping may be more appropriate than programs over-emphasizing a history of trauma exposure or the processing of a particular traumatic event. A recent analysis of data from 18 MSF non-specialized counselling programs (including the Chechnya program) demonstrates the relative strength of a flexible, trauma-informed treatment approach. Researchers found that although the MSF programs are originally designed for those affected by conflict and violence and appear to be effective in reducing symptoms, fewer than 20 % of cases were focused on trauma-related symptoms and a quarter of those seeking treatment did not link any traumatic event to their main presenting complaint, whereas 25 % identified domestic discord or violence as the underlying event triggering their main complaint [31].

Frequent reports of aggression and irritability symptoms across mental health problems further suggest that counseling programs, regardless of the intended focus or target population, should be prepared to address problems related to explosive anger and aggression. Given the potential bidirectional relationship between these emotional problems and problems in the family, early identification and prevention programs aimed at strengthening family supports are warranted.

As formative research for the MSF outcome evaluation, this study led us to develop a number of Chechen-specific items for inclusion in the MSF mental health assessment tool regarding being “out of temper”, emotional load, “emotional blowing”, and physical and verbal aggressiveness. Previous research validating assessment instruments with Chechen asylum seekers also noted these symptoms and concluded that standard diagnostic tools were likely to underestimate post-trauma related symptomatology relative to clinical interviews in this population [20], highlighting the importance of culturally adapting measures. Findings from the MSF outcome evaluation will provide additional information about the importance of these symptoms in a treatment-seeking sample, and the effectiveness of the existing treatment program in reducing these symptoms.

The current study identified approaches to help-seeking for mental health problems in Chechnya that inform where people with mental health problems may enter the system of care. First, we observed a greater awareness of the psychiatry model than has been observed in similar studies elsewhere [25, 32], reflected in the tendency for free list respondents to recommend predominantly counselors and psychologists as KIs. While this is likely due in part to our sampling approach, the finding is not entirely surprising given a relatively well-established history of specialist psychiatry in former Soviet countries [33, 34]. Second, we identified the Islamic center as an important parallel system of care alongside psychiatric and psychological services, with an apparent potential for collaboration and referrals between mental health professional and Islamic healing services. It seems that while some people may choose to initially seek help from one service or the other, if that service proves to be ineffective they are likely to look to an alternative option. The information provided by KIs reflects the perspective that Islamic healing is not in opposition to a biomedical model [35], and that understanding Islamic teachings on illness can also inform care provided in other settings [36].

### Limitations

The placement of our study in the capital city was relevant for programmatic purposes, as it is one of the two main sites, together with mountain villages, where the MSF counseling program is implemented in Chechnya. Because Grozny was at the center of conflict during both Chechen wars, it is unlikely that our respondents were sheltered from the impact of the conflict. However, Grozny is also now the center for reconstruction and development, meaning that people living in this area may experience daily life and psychosocial stressors differently from those in more rural areas less impacted by the reconstruction efforts. We cannot presume that this research is generalizable to people in other parts of the Chechnya.

As is the case with much qualitative research, this study was conducted with a convenience sample of adult men and women representing key stakeholder perspectives. In particular, inclusion of a hospital-based participants is likely to produce results that over-emphasize medical complaints, which were frequently reported in the FL interviews. We felt that this was appropriate given that the aim of the study was to identify and explore mental health problems in the Chechen context within a limited scope of study, and that it was likely that people seeking medical care would reference mental health problems in free listing. Because of this convenience sampling approach, the frequency of responses cannot be assumed to represent the prevalence of a given problem within the larger population. Rather than emphasizing absolute frequency, we highlighted instead items that were reported repeatedly versus those that were only reported by one or two people.

Another limitation was the small number of follow-up KI interviews we were able to conduct due to scheduling issues and the short time frame for data collection. Because this study was primarily intended to provide data relevant to the design of an outcome evaluation, we do not assume that we have identified all the issues affecting this population, or that we have obtained a truly nuanced understanding of them, but rather that we have established a foundation on which to build future research. For example, the frequently discussed problem of substance use was beyond the scope of our study to examine in more detail as this problem had not been a primary focus of the counseling program and therefore was not a priority problem for inclusion in the outcome evaluation. Yet the frequent discussion of this problem may highlight a need to develop relevant interventions and indicates the benefit of future research to better understand these problems in the Chechen context. Finally, we were not able to include KI interviews with representatives from the Islamic center. In future studies it would be helpful to include perspectives from those who provide services at these centers.

## Conclusion

The aim of this study was to gain a better understanding of mental health and psychosocial problems currently affecting people in Grozny, Chechnya, from their own perspective. This included understanding the nature of these problems, the effects of these problems on individuals, families, and communities, as well as coping and help seeking behavior. The purpose of gathering this information was to inform the development of assessment instruments for an outcome evaluation study of the MSF counseling approach. By conducting a relatively brief qualitative study, we were able to use the acquired data to adjust question wording and examples, and also to add questions to assess locally relevant symptoms to assess aggression and “not adequate” behavior. We believe this as important example of good practice in using qualitative methods to help develop tools for mental health care evaluations. This process also produced data regarding daily functioning of Chechen adults for the creation of a local functioning assessment; a description of this work will be included in the publication of the outcome evaluation.

Because of the dearth of literature on mental health problems in Chechnya, we believe our findings, although limited, also have broader utility for informing research and programming in this context. Given the consistent reports of high levels of irritability, aggression, “emotional blowing”, and “not adequate” behavior, future research exploring the relationship between these symptoms of distress and relevant syndromes is warranted. Additionally, research should further explore the relative role of conflict-related exposures and proximal social stressors in contributing to mental health of the population in this setting, particularly focusing on chronic stressors that could be addressed through individual and community-level programs to improve population health.

## Abbreviations

CCD, cultural concepts of distress; DIME, design, implementation, monitoring, and evaluation; FL, free list; KI, key interviewee; MSF, *Médecins Sans Frontières* (Doctors without Borders); PTSD, post-traumatic stress disorder
